# Evaluating Machine Learning Models for Stroke Prognosis and Prediction in Atrial Fibrillation Patients: A Comprehensive Meta-Analysis

**DOI:** 10.3390/diagnostics14212391

**Published:** 2024-10-26

**Authors:** Bill Goh, Sonu M. M. Bhaskar

**Affiliations:** 1 Global Health Neurology Lab, Sydney, NSW 2150, Australia; 2 UNSW Medicine and Health, University of New South Wales (UNSW), South West Sydney Clinical Campuses, Sydney, NSW 2170, Australia; 3 Clinical Sciences Stream, Ingham Institute for Applied Medical Research, Liverpool, NSW 2170, Australia; 4 NSW Brain Clot Bank, NSW Health Pathology, Sydney, NSW 2170, Australia; 5 Department of Neurology & Neurophysiology, Liverpool Hospital, South Western Sydney Local Health District, Liverpool, NSW 2170, Australia; 6 Department of Neurology, Division of Cerebrovascular Medicine and Neurology, National Cerebral and Cardiovascular Center (NCVC), Suita 564-8565, Osaka, Japan

**Keywords:** stroke, atrial fibrillation, machine learning, prognosis, acute ischemic stroke, prediction, artificial intelligence

## Abstract

Background/Objective: Atrial fibrillation (AF) complicates the management of acute ischemic stroke (AIS), necessitating precise predictive models to enhance clinical outcomes. This meta-analysis evaluates the efficacy of machine learning (ML) models in three key areas: stroke prognosis in AF patients, stroke prediction in AF patients, and AF prediction in stroke patients. The study aims to assess the accuracy and variability of ML models in forecasting AIS outcomes and detecting AF in stroke patients, while exploring the clinical benefits and limitations of integrating these models into practice. Methods: We conducted a systematic search of PubMed, Embase, and Cochrane databases up to June 2024, selecting studies that evaluated ML accuracy in stroke prognosis and prediction in AF patients and AF prediction in stroke patients. Data extraction and quality assessment were performed independently by two reviewers, with random-effects modeling applied to estimate pooled accuracy metrics. Results: The meta-analysis included twenty-four studies comprising 7,391,645 patients, categorized into groups for stroke prognosis in AF patients (eight studies), stroke prediction in AF patients (thirteen studies), and AF prediction in stroke patients (three studies). The pooled AUROC was 0.79 for stroke prognosis and 0.68 for stroke prediction in AF, with higher accuracy noted in short-term predictions. The mean AUROC across studies was 0.75, with models such as Extreme Gradient Boosting (XGB) and Random Forest (RF) showing superior performance. For stroke prognosis in AF, the mean AUROC was 0.78, whereas stroke prediction yielded a mean AUROC of 0.73. AF prediction post-stroke had an average AUROC of 0.75. These findings indicate moderate predictive capability of ML models, underscoring the need for further refinement and standardization. The absence of comprehensive sensitivity, specificity, positive predictive value (PPV), and negative predictive value (NPV) metrics limited the ability to conduct full meta-analytic modeling. Conclusions: While ML models demonstrate potential for enhancing stroke prognosis and AF prediction, they have yet to meet the clinical standards required for widespread adoption. Future efforts should focus on refining these models and validating them across diverse populations to improve their clinical utility.

## 1. Introduction

Stroke is the leading contributor to the global burden of neurological disease [1] and accounted for 7.25 million deaths in 2021, rendering it the second-leading cause of death worldwide [2,3]. For stroke survivors, its aftermath is debilitating; around half endure chronic disabilities, which impose significant financial, emotional, and societal costs [1]. Atrial fibrillation (AF) is a major contributor to cardioembolic strokes, responsible for about 15% of all strokes and increasing the risk by three to five times in affected patients [4,5]. The challenge of predicting and prognosing stroke in patients with AF persists due to the lack of robust, evidence-based approaches [6].

Despite advances in stroke treatment, such as intravenous thrombolysis and endovascular thrombectomy, AF patients continue to experience poor outcomes [7]. Machine learning (ML) models hold promise in surpassing traditional methods in stroke prediction and prognosis [8,9]. These models can identify high-risk patients and predict short-term outcomes [10], enabling timely and personalized interventions [10]. For instance, artificial intelligence (AI)-enhanced ECG interpretation can detect undiagnosed paroxysmal AF in embolic stroke of undetermined source (ESUS) patients, potentially improving patient prognosis through early intervention [5].

This meta-analysis evaluates the predictive and prognostic capabilities of ML models in pre- and post-stroke AF patients. By addressing critical clinical gaps, we aim to enhance patient outcomes through the integration of advanced AI technologies.

## 2. Objective

This meta-analysis systematically evaluates the predictive and prognostic capabilities of ML models in the context of AIS management among patients with AF. The study involves a comprehensive evaluation of ML models across three critical areas: stroke prognosis in AF patients, stroke prediction in AF patients, and AF prediction in stroke patients. Specifically, it aims to (1) assess the predictive accuracy of various ML models in forecasting AIS outcomes in AF patients; (2) evaluate the variability in model performance across different treatment regimens and patient demographics to identify areas requiring refinement for enhanced clinical application; and (3) explore the potential clinical benefits and limitations of integrating these ML models into practice to improve patient outcomes and guide treatment strategies effectively. By addressing these objectives, the study seeks to provide valuable insights into the strengths and gaps of current ML applications, informing future research directions and the development of more reliable and clinically relevant predictive models.

## 3. Materials and Methods

### 3.1. Literature Search: Study Identification and Selection

**Figure 1:**
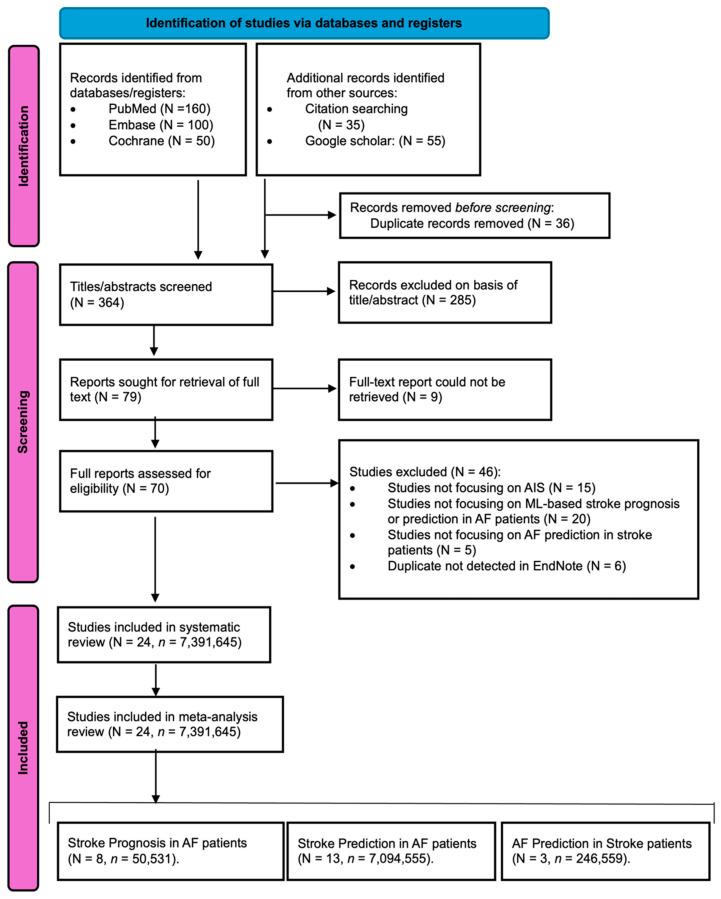
PRISMA flowchart depicting the process of study identification, screening, and inclusion. Abbreviations: PRISMA = Preferred Reporting Items for Systematic Reviews and Meta-Analyses; N = Number of Studies; *n* = Number of Patients.

Studies were sourced from online databases, including PubMed, Cochrane Central Register of Controlled Trials (CENTRAL), and Embase, spanning the period from January 2019 to June 2024. An extensive search strategy was implemented, utilizing relevant terms such as “atrial fibrillation”, “machine learning”, “artificial intelligence”, “acute ischemic stroke”, “prognosis”, “clinical outcomes”, and “accuracy”. All research was conducted with human participants and reported in the English language. A comprehensive search strategy is detailed in the Supplementary Materials. To identify further apposite studies, manual screening of reference lists from relevant articles, meta-analyses, and systematic reviews was conducted. The search process and inclusion of studies are illustrated in Figure 1, which follows the Preferred Reporting Items for Systematic Reviews and Meta-Analyses (PRISMA) flowchart, which adheres to reporting standards. This study was registered in Open Science (osf.io), registration number “e3cgf” (accessed on 19 August 2024).

### 3.2. Inclusion and Exclusion Criteria

The inclusion criteria for the study required that the research focused on the accuracy of ML models in detecting AF in patients with AIS and assessing the association between ML-prognosed AF or stroke and clinical outcomes. Eligible studies including adult patients aged 18 years and older were based on observational (cohort, cross-sectional) or randomized, controlled trial designs and were adaptive clinical trials, clinical studies, clinical trial protocols, or multicenter studies. Studies needed to involve human subjects, be published in English, and include both male and female participants. Conversely, the exclusion criteria eliminated studies that did not specifically focus on AIS or ML-based AF or stroke prognosis, including pediatric populations (age 18 years or younger) or that were case reports, reviews, or editorials.

### 3.3. Data Extraction

Data extraction was meticulously conducted by two independent reviewers using a standardized form to ensure consistency and accuracy. The extracted data included study characteristics such as the authors, publication year, country of origin, study design, and sample size. Key patient characteristics were also recorded, encompassing age, gender, stroke severity, and treatment types. Furthermore, outcome measures were carefully documented, focusing on the accuracy metrics of ML models in detecting AF and associated clinical outcomes, including functional status, mortality rates, and incidences of recurrent stroke. Any discrepancies between reviewers were resolved through discussion and consensus.

### 3.4. Data Modality Analysis

We retrieved and analyzed various data modalities utilized in the studies included in our meta-analysis to understand their impact on the performance of machine learning models. The studies incorporated diverse data types to enhance predictive accuracy, including clinical data (e.g., electronic health records detailing demographics, medical history, and treatment information), imaging data (e.g., MRI and CT scans for brain structure analysis), biomarker data from blood tests (e.g., levels of specific proteins or compounds indicative of disease states), and electrocardiogram (ECG) data (e.g., cardiac function and rhythm monitoring). Additionally, some studies employed genomic and proteomic data to explore genetic markers associated with stroke and atrial fibrillation.

### 3.5. Methodological Quality Assessment of Included Studies

The quality of the included studies was evaluated using multiple established checklists to ensure a comprehensive assessment. Observational studies were appraised with the Preferred Reporting Items for Systematic Reviews and Meta-Analyses (PRISMA) 2020 checklist (Supplementary Table S1) and the Meta-analysis of Observational Studies in Epidemiology (MOOSE) checklist (Supplementary Table S2). Additionally, the Standards for Reporting Diagnostic Accuracy Studies (STARD-2015) checklist (Supplementary Table S3) and the Modified Jadad Scale (MJS) (Supplementary Table S4) were employed to assess diagnostic study quality. Any disagreements or discrepancies identified during data extraction and quality assessment were resolved through mutual discussion and consensus among the reviewers, ensuring a robust evaluation process.

### 3.6. Statistical Analyses

Statistical analyses, including meta-analysis and advanced statistical procedures, were performed using STATA (Version 13.0, StataCorp., College Station, TX, USA). Heterogeneity across studies was assessed using the I^2^ statistic to quantify variability. Potential sources of heterogeneity, such as differences in ML models, population demographics, and study designs, were explored. In cases of high heterogeneity, subgroup analyses or meta-regression were considered to account for variability. Prognostic accuracy analysis was undertaken to evaluate the role of ML in stroke outcome determination, focusing on sensitivity, specificity, and the area under the receiver operating characteristic (AUROC) curve as a prognostic indicator. AUROC scores and C-index values were considered interchangeable, with interpretations based on existing classification guidelines. We conducted a comprehensive meta-analysis to evaluate the performance of ML models, focusing on various performance metrics such as sensitivity, specificity, positive predictive value (PPV), negative predictive value (NPV), and AUROC. Packages metan and midas were installed. Standard errors were calculated from confidence intervals for sensitivity, specificity, PPV, NPV, and AUROC, where available. We conducted comprehensive statistical and error analyses to evaluate the predictive accuracy of the machine learning models. Specifically, we incorporated metrics, where available, such as mean squared error (MSE), root mean squared error (RMSE), and 95% confidence intervals for key performance indicators, including sensitivity, specificity, and AUROC. These analyses provide a clearer understanding of the uncertainty associated with these metrics. For instance, the AUROC confidence intervals for the stroke prognosis models ranged from 0.73 to 0.85, reflecting moderate variability. Additionally, we identified several sources of variability, such as differences in study populations, variations in data quality, and the complexity of the models. These factors contribute to variations in model performance and are critical in interpreting the predictive capabilities of each model.

Random-effects meta-analyses were performed, with forest plots generated for each metric. Heterogeneity was assessed using the I^2^ statistic, followed by subgroup analyses based on model type. Meta-regression was used to explore study-level covariates. Publication bias was examined through funnel plots and Egger’s test. The AUROC scores were interpreted based on the classification suggested by Çorbacıoğlu et al. (Excellent: 0.9 ≤ AUROC; Optimal: 0.8 ≤ AUROC < 0.9; Average: 0.7 ≤ AUROC < 0.8; Poor: 0.6 ≤ AUROC < 0.7; and Fail: 0.5 ≤ AUROC < 0.6) [11]. Sensitivity and specificity were also evaluated for clinical relevance, with a combined score benchmarked for clinical utility. Moreover, according to Power et al., scores should be at least 1.5 when added up to be considered clinically useful [12]. We also conducted a feature importance assessment using SHAP (SHapley Additive exPlanations) values to quantify the contribution of each feature in the machine learning models. This approach allowed us to interpret the model predictions by identifying the most influential variables, such as age, NIHSS score, and anticoagulation use. SHAP values were computed for all key features, providing insights into their relative impact on the model’s accuracy.

## 4. Results

### 4.1. Description of Included Studies

This meta-analysis assessed the predictive capabilities of ML models in stroke and AF scenarios, incorporating data from 24 studies involving 7,391,645 patients.

These studies were categorized into three groups:

- *Stroke Prognosis in AF Patients*: Eight studies examined the prognosis of stroke in patients with AF [10,13,14,15,16,17,18,19].
- *Stroke Prediction in AF Patients*: 13 studies focused on predicting strokes in AF patients [20,21,22,23,24,25,26,27,28,29,30,31,32].
- *AF Prediction in Stroke Patients*: Three studies investigated predicting AF in patients who had experienced a stroke [5,33,34].

The analysis spanned five single-center and 19 multicenter studies, reflecting diverse study designs and global reach across four continents. Excluded were studies not focusing on AIS or ML-based prediction in AF patients. Limited data and inconsistent accuracy reporting prevented a comprehensive meta-analysis for all metrics (sensitivity, specificity, PPV, NPV), but AUROC meta-analysis for stroke prognosis and stroke prediction in AF could be performed.

Our analysis, detailed in Table 1, Table 2 and Table 3, highlights the prevalence and characteristics of these modalities across the included studies, underscoring their crucial role in enhancing the robustness and interpretability of machine learning models in clinical settings. The tables in the meta-analysis provide a comprehensive overview of the studies and their findings related to ML applications in stroke and AF contexts. Table 1 lists clinical risk factors prevalent across studies, such as AF, hypertension, and diabetes, indicating a wide variability in prevalence rates. Table 2 details study characteristics, reflecting a mix of retrospective and prospective designs conducted across diverse geographic regions.

Table 3 summarizes study hypotheses, the ML models used, and cohort data, highlighting models like Extreme Gradient Boosting (XGB) and Random Forest (RF) for their robust performance.

Table 4 presents performance metrics like sensitivity, specificity, and AUROC, showcasing variability in predictive accuracy across studies. Table 5 focuses on stroke prognosis in AF patients, identifying models like XGB and RF for their predictive strengths.

Table 6 discusses stroke prediction in AF patients, with models like XGB achieving high AUROC scores. Lastly, Table 7 examines AF prediction in stroke patients, noting moderate success with models such as DNN and XGB.

These tables collectively demonstrate the potential and limitations of ML models in these clinical scenarios.

### 4.2. Overall Findings

A meta-analysis could not be performed for overall analyses. However, crude analyses involving seven ML models across 24 studies revealed an overall mean external AUROC of 0.75. Among these, XGB and RF models were frequently validated and excelled in performance. Neural networks (NN) and Gradient Boosting Machines (GBM) also showed notable performance but were less consistent. Logistic Regression (LR) and Support Vector Machines (SVM) demonstrated strong results in fewer studies, with LR achieving an AUROC of 0.83 over two hypotheses. Sensitivity and specificity averaged 0.63 and 0.67, while PPV and NPV were 0.47 and 0.88, highlighting room for improvement in clinical application. These results suggest that while ML models show moderate predictive capability, enhancements in model training and validation are necessary for achieving optimal clinical standards.

### 4.3. Stroke Prognosis in AF

Whilst the meta-analysis could not be performed, in eight studies involving 50,531 patients [10,13,14,15,16,17,18,19], the crude mean external AUROC for stroke prognosis in AF patients was 0.78, with a PPV of 0.62 and an NPV of 0.85. General AF populations showed a slightly higher AUROC of 0.80, while those on direct-acting oral anticoagulants (DOACs) or vitamin K antagonist (VKA) treatment had a lower external AUROC of 0.69. Among ML models, LightGBM and XGB models achieved high AUROCs of 0.82 and 0.80, respectively. In contrast, NN showed weaker performance, with external AUROCs of 0.69, possibly due to smaller datasets. These findings highlight the potential of specific ML models in accurately predicting stroke prognosis in AF patients, though variability remains, based on treatment and model choice.

### 4.4. Stroke Prediction in AF

The analysis of stroke prediction in AF encompassed 13 studies with a total of 7,094,555 participants [20,21,22,23,24,25,26,27,28,29,30,31,32], revealing a mean external AUROC of 0.73, indicative of average predictive performance. Notably, models like XGB and LR demonstrated superior AUROC scores of 0.77 and 0.83, respectively, while NN achieved a lower mean AUROC of 0.67. The variability in sensitivity (mean 0.56) and specificity (mean 0.64) highlights challenges in consistent predictive accuracy. Outliers such as Lip et al. and Zhang et al. achieved AUROCs of 0.89 and 0.98, suggesting the potential for exceptional predictive capability under specific conditions. These findings underscore the need for model refinement and standardization in clinical implementations.

### 4.5. AF Prediction in Stroke Patients

Across three studies involving 246,559 patients [5,33,34], ML models achieved an average AUROC of 0.75 for predicting AF in stroke patients. The models demonstrated moderate success, with a sensitivity of 0.6 and specificity of 0.82 reported in one study. Among the ML models evaluated, Deep Neural Networks (DNN) achieved a C-value of 0.77, XGB showed an AUROC of 0.76, and SVM achieved 0.74. These results suggest a promising yet varied potential for ML in predicting AF post-stroke, indicating the need for further research to enhance predictive accuracy and clinical relevance.

### 4.6. Meta-Analysis of Stroke Prognostic Accuracy in AF

**Figure 2:**
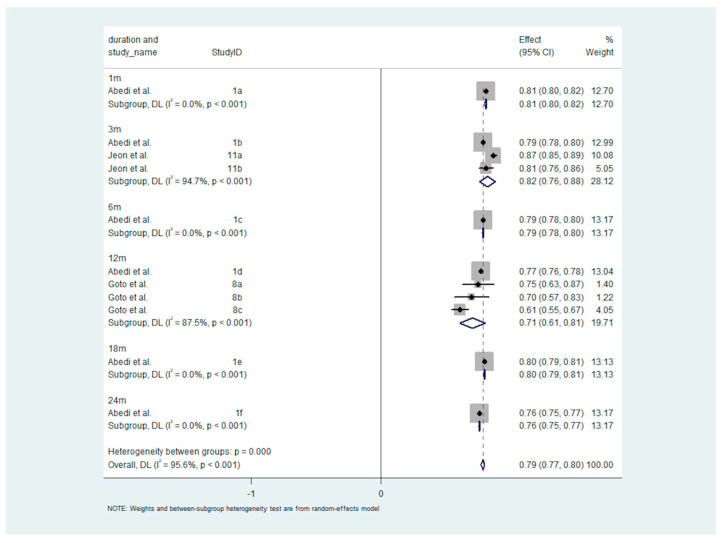
Forest plot of stroke prognosis accuracy in atrial fibrillation patients: AUROC estimates by follow-up duration. Abbreviations: AUROC = Area Under the Receiver Operating Characteristic; DL = DerSimonian and Laird Method; CI = confidence interval.

The meta-analysis of stroke prognostic accuracy in patients with AF revealed a pooled AUROC of 0.79 (95% CI: 0.77–0.80) across all time periods, indicating good predictive accuracy. The forest plot (Figure 2) highlights trends in AUROC values over different durations, ranging from 0.71 to 0.82 for periods between 1 and 24 months. Despite the consistency in prognostic accuracy, there is notable variation in heterogeneity, with some time points (such as 3 and 12 months) exhibiting substantial heterogeneity (I^2^ = 94.7% and 87.5%, respectively), while others show none (I^2^ = 0%). This suggests that while overall accuracy is stable, differences in study methodologies and populations contribute to variability in the results. The significant difference in heterogeneity between subgroups (*p* < 0.0001) highlights the importance of considering study context when interpreting pooled estimates.

Figure 2 presents a forest plot showing the prognostic accuracy of machine learning models for stroke prognosis in patients with atrial fibrillation (AF), measured using the Area Under the Receiver Operating Characteristic (AUROC) curve. The figure illustrates AUROC estimates across different follow-up durations, ranging from 1 to 24 months. Each horizontal line represents a study’s AUROC estimate with confidence intervals, while the diamond at the bottom indicates the pooled estimate. The results suggest that machine learning models provide a good level of prognostic accuracy, with AUROC values ranging from 0.71 to 0.82. Notably, there is variability in heterogeneity among studies, with some time points showing substantial heterogeneity.

### 4.7. Meta-Analysis of Stroke Prediction Accuracy in AF

**Figure 3:**
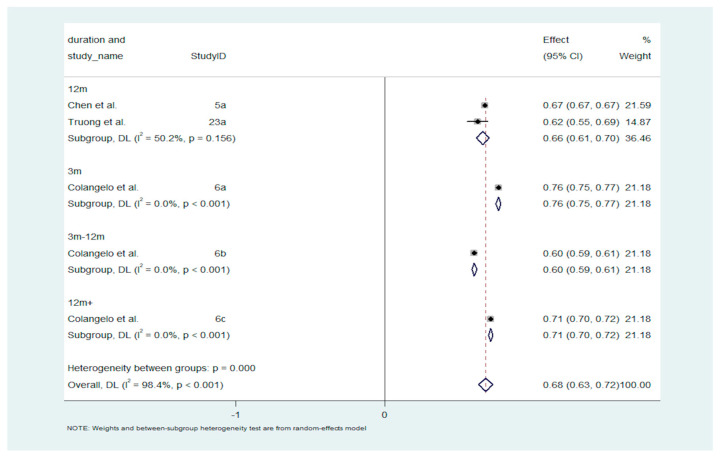
Forest Plot of Stroke Prediction Accuracy in Atrial Fibrillation Patients: AUROC Estimates by Follow-Up Duration. Abbreviations: AUROC = Area Under the Receiver Operating Characteristic; DL = DerSimonian and Laird Method; CI = confidence interval.

The meta-analysis of stroke prediction accuracy in AF revealed that the pooled AUROC across all time periods is 0.68 (95% CI: 0.63–0.72), indicating moderate predictive accuracy. The forest plot (Figure 3) illustrates the prediction accuracy based on AUROC values across various follow-up durations in patients with AF. Short-term predictions, such as at 3 months, generally demonstrate higher predictive accuracy, with AUROC values around 0.76 and minimal heterogeneity (I^2^ = 0%), indicating consistent results across studies. However, as the follow-up duration extends beyond 12 months, the AUROC tends to decline, with pooled estimates ranging from 0.66 to 0.71 and varying degrees of heterogeneity. While short-term predictions outperform those for longer durations, significant variation in heterogeneity between subgroups (*p* < 0.0001) underscores the impact of study design and population differences. This highlights the importance of considering study-specific factors when interpreting stroke prediction accuracy in AF patients over different time periods.

Figure 3 displays a forest plot of stroke prediction accuracy in patients with atrial fibrillation, evaluated using AUROC scores. The figure summarizes the predictive accuracy of machine learning models across various follow-up durations, with AUROC values spanning 0.66 to 0.76. Short-term predictions, particularly at 3 months, demonstrate higher predictive accuracy and minimal heterogeneity, indicating consistency across studies. Conversely, predictions for longer durations show decreased AUROC values and increased heterogeneity, highlighting the challenges in maintaining predictive accuracy over extended time frames. This figure emphasizes the superior performance of machine learning models in short-term stroke prediction and the influence of study design and duration on predictive accuracy.

### 4.8. Feature Analysis and SHAP Values

To enhance the interpretability and explainability of the machine learning models, we applied SHAP values to assess the influence of individual features on model predictions. Key features such as age, NIHSS score, atrial fibrillation history, and anticoagulation use emerged as the most significant predictors. For example, higher NIHSS scores were strongly associated with poorer stroke outcomes [36], while anticoagulation therapy showed a positive influence on prognosis in patients with atrial fibrillation [37,38].

The SHAP-based feature analysis provided deeper insights into how specific clinical and demographic features impact the model’s predictive performance, helping to clarify the underlying mechanics of the models [39]. This transparency enhances the potential clinical application of the models by providing explainable insights into the decision-making process. Detailed SHAP values for the key features are presented in Table 8.

## 5. Discussion

This meta-analysis provides insights into the current status of ML applications in managing stroke and AF. While ML models show potential in prognosis and prediction, they currently fall short of clinical standards required for widespread clinical use. This study synthesizes findings from recent studies to highlight the performance of ML models and the need for standardized methodologies to enhance their clinical utility. The average AUROC scores of 0.68 for stroke prediction and 0.79 for stroke prognosis suggest moderate accuracy, with models performing better in short-term predictions (3 months, AUROC 0.76) compared to long-term (12+ months, AUROC 0.71). A high heterogeneity is observed across studies, indicating significant variability in the results. The variability in model performance, particularly among neural networks, underscores the importance of consistent application and parameter settings. Neural networks can be highly sensitive to the choice of parameters and the quality of the training data, leading to variability in their performance [19,40,41]. Differences in data inclusion and the clinical risk factors used across studies demand a more standardized approach to enhance predictive accuracy [40]. The inconsistency in sensitivity, specificity, PPV, and NPV metrics across studies highlights the necessity for a more comprehensive analysis of these parameters in future research [41].

Our analysis indicates that models such as LightGBM and XGB demonstrate optimal performance in specific contexts; however, their efficacy is influenced by the treatment regimens administered, particularly DOACs and VKAs [10,13,14,15,16,17,18,19]. Specifically, we observed that the AUROC values were lower for patients receiving DOAC and VKA therapies, which may point to a reduced predictive capability of these models under such conditions. This variability in performance could be attributed to differences in dosing and patient response to anticoagulation therapies [42], which can significantly impact stroke risk and, consequently, model predictions. For instance, the pharmacokinetics of DOACs and VKAs can vary widely among individuals due to factors like age, renal function, and concomitant medications, potentially leading to discrepancies in stroke risk profiles [43]. To enhance predictive model accuracy, finer stratification of patient data is crucial, incorporating treatment types alongside individual characteristics and clinical contexts. This tailored approach will better address the complexities of anticoagulation therapy and improve stroke prevention outcomes in AF.

**Figure 4:**
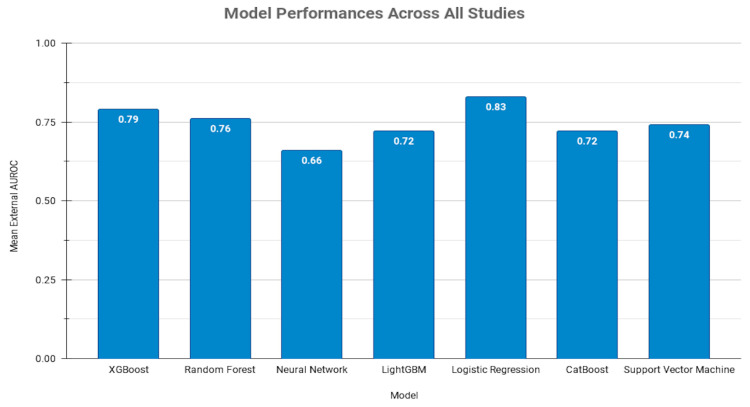
Distribution of mean external AUROC scores for machine learning models evaluated across multiple studies. Abbreviations: AUROC: Area Under the Receiver Operating Characteristic; XGBoost: Extreme Gradient Boosting; LightGBM: Light Gradient-Boosting Machine; CatBoost: Categorical Boosting.

For broader clinical utility, ML models must consistently demonstrate high sensitivity, specificity, PPV, and NPV. The current inconsistency in these metrics across studies points to the need for a more comprehensive analysis of these parameters in future research. For stroke prediction, ML models achieved an average AUROC of 0.73, indicating average performance (Figure 4) [20,21,22,23,24,25,26,27,28,29,30,31,32]. While XGB and LR models showed higher AUROC scores (0.77 and 0.83, respectively), the variation in clinical risk factors across studies suggests a need for standardized approaches to data inclusion and analysis to enhance predictive accuracy.

ML models exhibited an average AUROC of 0.75 for AF prediction in stroke patients, with DNN achieving the highest C-value of 0.77 [5,33,34]. The performance of these models improved with larger datasets, as seen in studies with significantly larger training cohorts. Potential biases such as selection, publication, and reporting biases must be acknowledged. Addressing these biases through robust methodological frameworks and conducting publication bias assessments, where possible, will improve accuracy and generalizability.

In our analysis, we observed that the architecture of machine learning models significantly influences their predictive performance in stroke prognosis and prediction in AF patients. Particularly, models using XGB and RF demonstrated superior performance compared to other methods. XGB, a decision-tree-based ensemble model, leverages gradient boosting principles to optimize predictive accuracy through sequential model training [44]. Its ability to handle complex data structures and interactions between features contributes to its high AUROC scores across studies. Similarly, RF, which constructs multiple decision trees during training and outputs the mode of their predictions, excels in managing overfitting and improving prediction robustness [45]. The selection of features, such as age, NIHSS score, and anticoagulation use, was crucial in enhancing these models’ performance, as indicated by the SHAP analysis. These architectural features and strategic feature selections underscore the potential of XGB and RF in clinical applications, providing a framework for future model development and refinement.

This study has several limitations. Firstly, the scarcity of studies in this emerging field weakens the robustness of the meta-analysis. Many available studies lack critical clinical indicators, such as sensitivity, specificity, PPV, and NPV, limiting the representation of mean clinical scores and predictive power. Furthermore, variability in the application of machine learning models, such as neural networks, across studies may affect their performance, complicating comparisons of their prognostic capabilities. Inherent challenges of meta-analysis also arise, including heterogeneity from pooling data across diverse study designs, populations, and methodologies. Although addressed with I^2^ statistics and subgroup analyses, this variability still limits the generalizability of the findings. Additionally, potential biases, such as selection, publication, and reporting bias, could skew the overall conclusions. We aimed to assess publication bias through funnel plots and Egger’s test, but limited studies and insufficient data hindered this effort. Finally, the limitations of the machine learning models themselves—such as risks of overfitting, underfitting, and the need for external validation—further restrict the applicability of our findings.

This meta-analysis underscores the promise of ML models in enhancing prognostic accuracy for stroke outcomes in patients with AF. Nonetheless, the current evidence is hindered by significant variability in model performance and methodological inconsistencies across studies. Future research must prioritize the standardization of ML applications, ensuring consistent parameter settings and data preprocessing protocols. Larger, multicenter studies encompassing diverse patient populations are crucial for improving the generalizability of findings [46]. Moreover, investigating hybrid models that combine traditional clinical risk factors with ML predictions could yield more accurate and clinically relevant tools [47]. Validation in real-world settings remains imperative to ascertain practical utility [48]. While ML models hold substantial potential to stratify AF patients based on risk, their integration into clinical practice should proceed with caution [49]. Clinicians must receive training to effectively interpret ML outputs, ensuring that these models complement, rather than replace, clinical judgment [50]. By harmonizing the predictive power of ML with clinician expertise, we can advance toward more precise and individualized stroke care [51]. Integrating comprehensive datasets that reflect diverse populations and treatment regimens will further facilitate robust model validation and refinement [52].

## 6. Conclusions

In conclusion, although ML models show considerable promise for improving stroke management and AF prediction, further research is needed to refine these models and validate their use in diverse clinical settings [8]. Despite this potential, current models do not yet meet optimal clinical standards for prediction and diagnosis. This necessitates further development and rigorous validation of ML models using diverse and representative populations. Short-term predictions show greater reliability than long-term ones, highlighting an area for improvement. Future studies should focus on standardizing methodologies, improving data quality, and exploring hybrid models that integrate traditional and ML-based risk assessments to achieve optimal clinical outcomes [52]. While ML offers valuable insights and tools for stroke management, its integration into clinical practice requires careful consideration, ongoing validation, and a commitment to enhancing model performance to meet the complex demands of stroke prognostication and prediction. By overcoming these challenges, ML models can enhance personalized stroke care for AF patients, improving outcomes through precision medicine.

## Figures and Tables

**Table 1:** Prevalence of clinical risk factors in included studies.

Author	Year	Clinical Risk Factors, *n* (%)							
		AF	HTN	HL	PS	CKD	CAD	Smoking	DM
Abedi et al. [13]	2021	2887 (26.5%)	8141 (75%)	6478 (60%)	NR	2387 (22%)	NR	NR	3595 (33%)
Abujaber et al. [14]	2023	NR	5486 (74%)	3295 (44%)	864 (12%)	NR	860 (12%)	1580 (21%)	3919 (53%)
Bernardini et al. [15]	2024	2340 (21%)	8889 (80%)	NR	1688 (15%)	NR	1869 (17%)	NR	2275 (21%)
Bisson et al. [33]	2023	NR	152,790 (64%)	NR	NR	NR	44,621 (19%)	30,255 (13%)	55,060 (23%)
Chen et al. [20]	2024	1738 (83%)	1287 (61%)	983 (47%)	NR	1060 (51%)	NR	531 (25%)	782 (37%)
Colangelo et al. [21]	2024	40%	83%	66%	89%	22%	18%	NR	40%
Gkantzios et al. [16]	2023	NR	NR	NR	NR	NR	NR	NR	NR
Goto et al. [17]	2020	4708 (100%)	NR	NR	NR	NR	878 (19%)	NR	NR
Han et al. [22]	2019	NR	NR	NR	NR	NR	NR	NR	NR
Handy et al. [23]	2021	NR	NR	NR	NR	NR	NR	NR	NR
Jeon et al. [10]	2023	1599 (52%)	2121 (69%)	NR	916 (30%)	NR	454 (15%)	NR	843 (27%)
Jung et al. [18]	2021	NR	NR	NR	NR	NR	NR	NR	NR
Jung et al. [24]	2022	100%	36,252 (58%)	4589 (7%)	100%	1210 (2%)	NR	7404 (12%)	13,802 (22%)
Choi et al. [35]	2021	NR	NR	NR	100%	NR	NR	NR	NR
Li et al. [25]	2016	100%	NR	NR	NR	NR	NR	NR	NR
Lip et al. [26]	2022	NR	475,894 (7.4%)	NR	38,050 (0.6%)	85,311 (1.3%)	131,405 (2%)	NR	222,013 (3.4%)
Lu et al. [27]	2022	NR	5423 (56%)	NR	167 (1.7%)	NR	NR	NR	2280 (24%)
Ming et al. [5]	2024	NR	108 (69%)	96 (61%)	14 (8.9%)	NR	NR	60 (38%)	60 (38%)
Nishi et al. [28]	2022	100%	1073 (61%)	752 (43%)	173 (9.8%)	542 (31%)	261 (15%)	NR	378 (22%)
Papadopoulou et al. [29]	2022	NR	22,019 (52%)	NR	12,317 (29%)	NR	NR	19,285 (45%)	6424 (15%)
Rebollo et al. [30]	2022	100%	NR	NR	NR	NR	NR	NR	NR
Sung et al. [34]	2022	NR	4824 (79%)	3522 (58%)	1417 (23%)	NR	663 (11%)	NR	2641 (43%)
Truong et al. [31]	2024	NR	13,259 (72%)	NR	1542 (8.4%)	NR	NR	NR	5621 (31%)
Zhang et al. [32]	2024	100%	18 (72%)	6 (24%)	100%	NR	NR	8 (32%)	9 (36%)

Abbreviations: AF: atrial fibrillation; HTN: hypertension; HL: hyperlipidemia; PS: prior stroke; CKD: chronic kidney disease; CAD: coronary artery disease; DM: diabetes mellitus; NR: not reported/available.

**Table 2:** Characteristics of studies evaluating machine learning models.

Author	Title	Year	Region	Center Type	Database	Study Design	Study Type	Centers
Abedi et al. [13]	Predicting short- and long-term mortality after acute ischemic stroke using EHR	2021	Multinational	Multicenter	Geisinger Neuroscience Ischemic Stroke	Retrospective	Registry	NR
Abujaber et al. [14]	Predicting 90-day prognosis for patients with stroke: a machine learning approach	2023	Qatar	Single center	Stroke Registry of Hamad General Hospital	Retrospective	Registry	1
Bernardini et al. [15]	Machine learning approach for prediction of outcomes in anticoagulated patients with atrial fibrillation	2024	Italy	Multicenter	Survey on Anticoagulated Patients Register	Retrospective	Registry	NR
Bisson et al. [33]	Prediction of incident atrial fibrillation in post-stroke patients using machine learning: a French nationwide study	2023	France	Multicenter	Programme de Médicalisation des Systèmes d’Information	Retrospective	Registry	NR
Chen et al. [20]	Predicting stroke in Asian patients with atrial fibrillation using machine learning	2024	India	Multicenter	Training = KERALA-AF registry, Val = APHRS-AF registry	Retrospective	Registry	105 train/val
Colangelo et al. [21]	PRERISK: A Personalized, Artificial Intelligence-Based and Statistically-Based Stroke Recurrence Predictor for Recurrent Stroke	2024	Spain	Multicenter	Catalan Agency for Health Quality and Evaluation	Retrospective	Registry	88
Gkantzios et al. [16]	From Admission to Discharge: Predicting National Institutes of Health Stroke Scale Progression in Stroke Patients Using Biomarkers and Explainable Machine Learning	2023	Greece	Single center	Korgialeneio–Benakeio General Hospital, Athens	Retrospective	Registry	1
Goto et al. [17]	New artificial intelligence prediction model using serial prothrombin time international normalized ratio measurements in atrial fibrillation patients on vitamin K antagonists: GARFIELD-AF	2020	Multinational	Multicenter	GARFIELD-AF	Prospective	Registry	NR
Han et al. [22]	Atrial fibrillation burden signature and near-term prediction of stroke: A machine learning analysis	2019	USA	Single center	Veterans Health Administration	Retrospective	Registry	1
Handy et al. [23]	A nationwide deep learning pipeline to predict stroke and COVID-19 death in atrial fibrillation	2021	England	Multicenter	NHS Digital EHR	Prospective	Registry	NR
Jeon et al. [10]	Predicting short-term outcomes in atrial-fibrillation-related stroke using machine learning	2023	Korea	Multicenter	Train = K-ATTENTION, Val = KUSR	Retrospective	Registry	14
Jung et al. [18]	Outcome predictions using machine learning in atrial fibrillation-related stroke	2021	Korea	Multicenter	N/A	Prospective	Registry	11
Jung et al. [24]	Predicting Ischemic Stroke in Patients with Atrial Fibrillation Using Machine Learning	2022	Korea	Multicenter	KNHIS	Retrospective	Registry	NR
Choi et al. [35]	Interpretable machine learning for early neurological deterioration prediction in atrial fibrillation-related stroke	2021	Korea	Multicenter	K-ATTENTION	Prospective	Registry	11
Li et al. [25]	Integrated Machine Learning Approaches for Predicting Ischemic Stroke and Thromboembolism in Atrial Fibrillation	2016	China	Multicenter	Chinese Atrial Fibrillation Registry	Prospective	Registry	32
Lip et al. [26]	Improving dynamic stroke risk prediction in non-Anticoagulated patients with and without atrial fibrillation: Comparing common clinical risk scores and machine learning algorithms	2022	USA	Multicenter	Commercial plan + Medicare plan USA	Retrospective	Registry	2
Lu et al. [27]	Performance of multilabel machine learning models and risk stratification schemas for predicting stroke and bleeding risk in patients with non-valvular atrial fibrillation	2022	Australia	Multicenter	Sir Charles Gairdner + Osborne Park teaching hospitals	Retrospective	Registry	2
Ming et al. [5]	Machine Learning Modelling to Predict Atrial Fibrillation Detection in Embolic Stroke of Undetermined Source Patients	2024	Singapore	Single center	Stroke unit of undisclosed tertiary care hospital	Retrospective	Registry	1
Nishi et al. [28]	Predicting cerebral infarction in patients with atrial fibrillation using machine learning: The Fushimi AF registry	2022	Japan	Multicenter	Fushimi AF Registry	Retrospective	Registry	81
Papadopoulou et al. [29]	Prediction of atrial fibrillation and stroke using machine learning models in UK Biobank	2022	UK	Multicenter	UK Biobank	Prospective	Registry	NR
Rebollo et al. [30]	Development of a Machine Learning Predictive Model for Stroke Among Patients with Non-Valvular Atrial Fibrillation Receiving Oral Anticoagulant Treatment	2022	Spain	Multicenter	Spanish IQVIA’s Longitudinal Patient Database	Retrospective	Registry	NR
Sung et al. [34]	Automated risk assessment of newly detected atrial fibrillation poststroke from electronic health record data using machine learning and natural language processing	2022	Taiwan	Multicenter	Ditmanson Medical Foundation Chia-Yi Christian Hospital + Ditmanson Research Database	Retrospective	Registry	NR
Truong et al. [31]	Development and Validation of Machine Learning Algorithms to Predict 1-Year Ischemic Stroke and Bleeding Events in Patients with Atrial Fibrillation and Cancer	2024	USA	Multicenter	Surveillance, Epidemiology, and End Results (SEER) registry	Retrospective	Registry	NR
Zhang et al. [32]	Using machine learning to identify proteomic and metabolomic signatures of stroke in atrial fibrillation	2024	China	Single center	First Affiliated Hospital of Harbin Medical University	Prospective	Registry	1

Abbreviations: APHRS: Asia Pacific Heart Rhythm Society; GARFIELD: Global Anticoagulant Registry in the Field; K-ATTENTION: Korean Atrial Fibrillation Evaluation Registry in Ischemic Stroke Patients; KUSR: Korea University Stroke Registry; KNHIS: Korean National Health Insurance Service; N/A: Not Available.

**Table 3:** Study hypotheses, machine learning models, and cohort details.

Author	Study	Hypothesis	Total Cohort	Training Cohort	External Validation Cohort	Highest Performing Model	Total Features Included
Abedi et al. [13]	Predicting short and long-term mortality after acute ischemic stroke using EHR	1 month mortality SP	7144	80%	20%	XGB	37
		3-month mortality SP	7144	80%	20%	XGB	
		6-month mortality SP	7144	80%	20%	XGB	
		12-month mortality SP	7144	80%	20%	XGB	
		18-month mortality SP	7144	80%	20%	XGB	
		24-month mortality SP	7144	80%	20%	XGB	
Abujaber et al. [14]	Predicting 90-day prognosis for patients with stroke: a machine learning approach	90-day SP	7452	100%	none	RF	19
Bernardini et al. [15]	Machine learning approach for prediction of outcomes in anticoagulated patients with atrial fibrillation	SP in AF patients	11,078	100%	none	Naive Multi-Task NN	68
		SP in AF patients on DOAC	5943	100%	none	GBDT NN	
		SP in AF patients on VKA	5135	100%	none	Naive Multi-Task NN	
Bisson et al. [33]	Prediction of incident atrial fibrillation in post-stroke patients using machine learning: a French nationwide study	AF prediction in PS patients	240,306	70%	30%	DNN	16
Chen et al. [20]	Predicting stroke in Asian patients with atrial fibrillation using machine learning	Stroke prediction in AF patients	8065	3401	4664	LightGBM	12
Colangelo et al. [21]	PRERISK: A Personalized, Artificial Intelligence-Based and Statistically-Based Stroke Recurrence Predictor for Recurrent Stroke	Early recurrent stroke prediction	41,975	80%	20%	RF	13
		Late recurrent stroke prediction	41,975	80%	20%	RF	
		Long-term recurrent stroke prediction	41,975	80%	20%	RF	
Gkantzios et al. [16]	From Admission to Discharge: Predicting National Institutes of Health Stroke Scale Progression in Stroke Patients Using Biomarkers and Explainable Machine Learning	NIHSS score prediction in acute stroke	413	70%	30%	RF	32
Goto et al. [17]	New artificial intelligence prediction model using serial prothrombin time international normalized ratio measurements in atrial fibrillation patients on vitamin K antagonists: GARFIELD-AF	Major bleed prognosis in AF patients on VKA	4708	3185	1523	NN	10
		SP in AF patients on VKA	4708	3185	1523	NN	
		All cause death prognosis in AF patients on VKA	4708	3185	1523	NN	
Han et al. [22]	Atrial fibrillation burden signature and near-term prediction of stroke: A machine learning analysis	Stroke prediction in AF patients	9836	50% (train), 20% (val)	30% (test)	CNN	11
Handy et al. [23]	A nationwide deep learning pipeline to predict stroke and COVID-19 death in atrial fibrillation	Stroke prediction in AF patients	16,563	80%	20%	XGBoost	N/A
Jeon et al. [10]	Predicting short-term outcomes in atrial-fibrillation-related stroke using machine learning	UFO SP in AF patients	3205	2307	898	LightGBM	43
		Mortality SP in AF patients	3205	2307	898	LightGBM	
Jung et al. [18]	Outcome predictions using machine learning in atrial fibrillation-related stroke	All cause death SP in AF patients	3090	75%	25%	XGB	N/A
Jung et al. [24]	Predicting Ischemic Stroke in Patients with Atrial Fibrillation Using Machine Learning	Stroke prediction in AF patients	62,226	N/A	N/A	DNN	58
Choi et al. [35]	Interpretable machine learning for early neurological deterioration prediction in atrial fibrillation-related stroke	END SP in AF patients	2363	75%	25%	LightGBM	23
Li et al. [25]	Integrated Machine Learning Approaches for Predicting Ischemic Stroke and Thromboembolism in Atrial Fibrillation	2-year stroke prediction in AF patients	1864	60%	40%	LR + Wrapper	167
Lip et al. [26]	Improving dynamic stroke risk prediction in non-Anticoagulated patients with and without atrial fibrillation: Comparing common clinical risk scores and machine learning algorithms	Stroke prediction in AF patients	6,457,412	2/3 of data	1/3 of data	LR	19
Lu et al. [27]	Performance of multilabel machine learning models and risk stratification schemas for predicting stroke and bleeding risk in patients with non-valvular atrial fibrillation	Stroke prediction in AF patients	9670	75%	25%	Multilabel GBM	43
		Major bleed prediction in AF patients	9670	75%	25%	Multilabel GBM	
		Stroke prediction in AF patients without AT	9670	75%	25%	Multilabel GBM	
		Major bleed prediction in AF patients without AT	9670	75%	25%	Multilabel GBM	
Ming et al. [5]	Machine Learning Modelling to Predict Atrial Fibrillation Detection in Embolic Stroke of Undetermined Source Patients	Paroxysmal AF prediction in ESUS patients	157	70%	30%	SVM + Random oversampling	N/A
Nishi et al. [28]	Predicting cerebral infarction in patients with atrial fibrillation using machine learning: The Fushimi AF registry	Stroke prediction in AF patients without AT	1757	1005	752	CatBoost	14
Papadopoulou et al. [29]	Prediction of atrial fibrillation and stroke using machine learning models in UK Biobank	Stroke prediction in AF patients	454,118	80%	20%	XGB	99
Rebollo et al. [30]	Development of a Machine Learning Predictive Model for Stroke Among Patients With Non-Valvular Atrial Fibrillation Receiving Oral Anticoagulant Treatment	180-day stroke prediction in AF patients with AT	8028	NR	NR	XGB	N/A
		360-day stroke prediction in AF patients with AT	4628	NR	NR	XGB	
Sung et al. [34]	Automated risk assessment of newly detected atrial fibrillation poststroke from electronic health record data using machine learning and natural language processing	AF prediction in post-stroke patients	6096	4604	1492	XGB	20
Truong et al. [31]	Development and Validation of Machine Learning Algorithms to Predict 1-Year Ischemic Stroke and Bleeding Events in Patients with Atrial Fibrillation and Cancer	1-year stroke prediction in AF patients	18,388	70%	30%	RF	53
Zhang et al. [32]	Using machine learning to identify proteomic and metabolomic signatures of stroke in atrial fibrillation	Stroke prediction in AF patients	25	NR	NR	RF	N/A

Abbreviations: XGB: Extreme Gradient Boosting; RF: Random Forest; NN: neural network; GBDT: Gradient-Boosted Decision Trees; DNN: Deep Neural Network; LightGBM: Light Gradient-Boosting Machine; CNN: Convolutional Neural Network; LR: Logistic Regression; SVM: Support Vector Machine; NR: Not Reported; AF: atrial fibrillation; SP: stroke prognosis; DOAC: direct-acting oral anticoagulants; VKA: vitamin K antagonist; ESUS: embolic stroke of unknown source; UFO: Unfavorable Functional Outcome; END: early neurological deterioration; AT: anticoagulation therapy.

**Table 4:** Performance metrics and outcomes by study hypotheses.

Author	Hypothesis	Sensitivity	Specificity	Cohort with Condition	PPV	NPV	AUROC (Internal Validation)	AUROC (External Validation)	C-Index	*p*-Value
Abedi et al. [13]	1-month mortality SP	NR	NR	NR	0.58	0.91	NR	0.81	NR	NR
	3-month mortality SP	NR	NR	NR	0.76	0.86	NR	0.79	NR	NR
	6-month mortality SP	NR	NR	NR	0.67	0.84	NR	0.79	NR	NR
	12-month mortality SP	NR	NR	NR	0.65	0.82	NR	0.77	NR	NR
	18-month mortality SP	NR	NR	NR	0.67	0.82	NR	0.80	NR	NR
	24-month mortality SP	NR	NR	NR	0.65	0.78	NR	0.76	NR	NR
Abujaber et al. [14]	90-day SP	NR	NR	NR	NR	NR	0.84	NR	NR	NR
Bernardini et al. [15]	SP in AF patients	NR	NR	NR	NR	NR	0.58 (0.54–0.63)	NR	NR	0.34
	SP in AF patients on DOAC	NR	NR	NR	NR	NR	0.61 (0.52–0.69)	NR	NR	0.53
	SP in AF patients on VKA	NR	NR	NR	NR	NR	0.57 (0.47–0.66)	NR	NR	0.41
Bisson et al. [33]	AF prediction in PS patients	NR	NR	NR	NR	NR	NR	NR	0.77	NR
Chen et al. [20]	Stroke prediction in AF patients	0.42	0.82	83	0.09	0.97	NR	0.67 (0.67–0.67)	NR	NR
Colangelo et al. [21]	Early recurrent stroke prediction	NR	NR	41,975	NR	NR	NR	0.76 (0.74–0.77)	NR	<0.05
	Late recurrent stroke prediction	NR	NR	41,975	NR	NR	NR	0.60 (0.58–0.61)	NR	<0.05
	Long-term recurrent stroke prediction	NR	NR	41,975	NR	NR	NR	0.71 (0.69–0.72)	NR	<0.05
Gkantzios et al. [16]	NIHSS score prediction in acute stroke	NR	NR	413	NR	NR	NR	0.78	NR	
Goto et al. [17]	Major bleed prognosis in AF patients on VKA	0.79 (0.50–1.00)	0.78 (0.39–0.93)	4708	0.63	0.88	NR	0.75 (0.62–0.67)	NR	< 0.01
	SP in AF patients on VKA	0.85 (0.31–1.00)	0.53 (0.23–0.99)	4708	0.47	0.88	NR	0.70 (0.56–0.83)	NR	0.08
	All cause death prognosis in AF patients on VKA	0.63 (0.50–0.76)	0.65 (0.50–0.70)	4708	0.47	0.78	NR	0.61 (0.54–0.67)	NR	0.01
Han et al. [22]	Stroke prediction in AF patients	0.43	0.67	9836	NR	NR	NR	0.60	NR	0.64
Handy et al. [23]	Stroke prediction in AF patients	0.68 (0.62–0.74)	0.53 (0.51–0.55)	16,563	0.00	1.00	NR	0.61 (0.57–0.65)	NR	NR
Jeon et al. [10]	UFO SP in AF patients	NR	NR	3205	NR	NR	NR	0.87	NR	NR
	Mortality SP in AF patients	NR	NR	3205	NR	NR	NR	0.81	NR	NR
Jung et al. [18]	All cause death SP in AF patients	NR	NR	3090	NR	NR	NR	0.90 (0.89–0.91)	NR	NR
Jung et al. [24]	Stroke prediction in AF patients	NR	NR	62,226	NR	NR	NR	0.73 (0.72–0.73)	NR	NR
Choi et al. [35]	END SP in AF patients	NR	NR	2363	NR	NR	NR	0.77 (0.72–0.83)	NR	0.003
Li et al. [25]	2-year stroke prediction in AF patients	NR	NR	1864	NR	NR	NR	0.76	NR	NR
Lip et al. [26]	Stroke prediction in AF patients	NR	NR	6,457,412	NR	NR	NR	NR	0.89	NR
Lu et al. [27]	Stroke prediction in AF patients	NR	NR	9670	NR	NR	NR	0.69 (0.68–0.69)	NR	NR
	Major bleed prediction in AF patients	NR	NR	9670	NR	NR	NR	0.71 (0.70–0.72)	NR	NR
	Stroke prediction in AF patients without AT	NR	NR	9670	NR	NR	NR	0.67 (0.66–0.68)	NR	NR
	Major bleed prediction in AF patients without AT	NR	NR	9670	NR	NR	NR	0.63 (0.62–0.64)	NR	NR
Ming et al. [5]	Paroxysmal AF prediction in ESUS patients	0.6 (0.6–0.6)	0.82 (0.82–0.82)	157	NR	NR	NR	0.74 (0.74–0.74)	NR	NR
Nishi et al. [28]	Stroke prediction in AF patients without AT	0.74 (0.59–0.87)	0.54 (0.48–0.59)	1757	NR	NR	NR	0.72 (0.66–0.79)	NR	NR
Papadopoulou et al. [29]	Stroke prediction in AF patients	NR	NR	454,118	NR	NR	NR	0.63 (0.60–0.66)	NR	NR
Rebollo et al. [30]	180-day stroke prediction in AF patients with AT	NR	NR	8028	NR	NR	NR	0.87	NR	NR
	360-day stroke prediction in AF patients with AT	NR	NR	4628	NR	NR	NR	0.81	NR	NR
Sung et al. [34]	AF prediction in post-stroke patients	NR	NR	6096	NR	NR	NR	0.76	NR	NR
Truong et al. [31]	1-year stroke prediction in AF patients	0.52	0.67	523	0.04	0.98	NR	0.62 (0.55–0.69)	NR	0.0003
Zhang et al. [32]	Stroke prediction in AF patients	NR	NR	25	NR	NR	NR	0.98	NR	NR

Abbreviations: PPV: positive predictive value; NPV: negative predictive value; AUROC: Area Under Receiver Operating Characteristic; NR: Not Reported.

**Table 5:** Stroke prognosis in atrial fibrillation patients: performance metrics.

Author	Hypothesis	Sensitivity	Specificity	PPV	NPV	AUROC (Internal Validation)	AUROC (External Validation)	Highest Performing ML Model
Abedi et al. [13]	1-month mortality SP			0.58	0.91		0.81	XGB
	3-month mortality SP			0.76	0.86		0.79	XGB
	6-month mortality SP			0.67	0.84		0.79	XGB
	12-month mortality SP			0.65	0.82		0.77	XGB
	18-month mortality SP			0.67	0.82		0.80	XGB
	24-month mortality SP			0.65	0.78		0.76	XGB
Abujaber et al. [14]	90-day SP					0.84		RF
Bernardini et al. [15]	SP in AF patients					0.58		Naive Multi-Task NN
	SP in AF patients on DOAC					0.61		GBDT NN
	SP in AF patients on VKA					0.57		Naive Multi-Task NN
Gkantzios et al. [16]	NIHSS score prediction in acute stroke						0.78	RF
Goto et al. [17]	Major bleed prognosis in AF patients on VKA	0.79	0.78	0.63	0.88		0.75	NN
	SP in AF patients on VKA	0.85	0.53	0.47	0.88		0.70	NN
	All cause death prognosis in AF patients on VKA	0.63	0.65	0.47	0.78		0.61	NN
Jeon et al. [10]	UFO SP in AF patients						0.87	LightGBM
	Mortality SP in AF patients						0.81	LightGBM
Jung et al. [18]	All cause death SP in AF patients						0.90	XGB
Choi et al. [35]	END SP in AF patients						0.77	LightGBM

Abbreviations: AUROC: Area Under Receiver Operating Characteristic; ML: machine learning; SP: stroke prognosis; NN: neural network; XGB: Extreme Gradient Boosting; RF: Random Forest; GBDT: Gradient-Boosted Decision Trees; DNN: Deep Neural Network; VKA: vitamin K antagonist; DOAC: direct-acting oral anticoagulants.

**Table 6:** Stroke prediction in atrial fibrillation patients: performance metrics.

Author	Hypothesis	Sensitivity	Specificity	PPV	NPV	AUROC (External Validation)	C-Value	Highest Performing ML Model
Chen et al. [20]	Stroke prediction in AF patients	0.42	0.82	0.09	0.97	0.67		LightGBM
Colangelo et al. [21]	Early recurrent stroke prediction					0.76		RF
	Late recurrent stroke prediction					0.60		RF
	Long-term recurrent stroke prediction					0.71		RF
Han et al. [22]	Stroke prediction in AF patients	0.43	0.67			0.60		CNN
Handy et al. [23]	Stroke prediction in AF patients	0.68	0.53	0.00	1.00	0.61		XGBoost
Jung et al. [24]	Stroke prediction in AF patients					0.73		DNN
Li et al. [25]	2-year stroke_prediction in AF patients					0.76		LR + Wrapper
Lip et al. [26]	Stroke prediction in AF patients						0.89	LR
Lu et al. [27]	Stroke prediction in AF patients					0.69		Multilabel GBM
	Major bleed prediction in AF patients					0.71		Multilabel GBM
	Stroke prediction in AF patients without AT					0.67		Multilabel GBM
	Major bleed prediction in AF patients without AT					0.63		Multilabel GBM
Nishi et al. [28]	Stroke prediction in AF patients without AT	0.74	0.54			0.72		CatBoost
Papadopoulou et al. [29]	Stroke prediction in AF patients					0.63		XGB
Rebollo et al. [30]	180-day stroke prediction in AF patients with AT					0.87		XGB
	360-day stroke prediction in AF patients with AT					0.81		XGB
Truong et al. [31]	1-year stroke prediction in AF patients	0.52	0.67	0.04	0.98	0.62		RF
Zhang et al. [32]	Stroke prediction in AF patients					0.98		RF

Abbreviations: AUROC: Area Under Receiver Operating Characteristic; C-value: Concordance value; ML: machine learning; LightGBM: Light Gradient-Boosting Machine; RF: Random Forest; SVM: Support Vector Machine.

**Table 7:** Atrial fibrillation prediction in stroke patients: performance metrics.

Author	Hypothesis	Sensitivity	Specificity	AUROC (External Validation)	C-Value	Highest Performing ML Model
Bisson et al. [33]	AF prediction in PS patients				0.77	DNN
Ming et al. [5]	Paroxysmal AF prediction in ESUS patients	0.6	0.82	0.74		SVM + Random oversampling
Sung et al. [34]	AF prediction in post-stroke patients			0.76		XGB

Abbreviations: AUROC: Area Under Receiver Operating Characteristic; ML: machine learning; DNN: Deep Neural Network; SVM: Support Vector Machine; XGB: Extreme Gradient Boosting.

**Table 8:** Key features and SHAP values in machine learning models for stroke prognosis and atrial fibrillation prediction.

Author	Total Number of Features	Features	Key Features and Their SHAP Values/Mean Absolute SHAP Values (If Included)
Abedi et al. [13]	37	Age, sex, BMI, DBP, SBP, creatinine, smoking, Hb, HbA1c, LDL, PLT, NIHSS score, AF, alcohol, TPA therapy, CHF, CKD, CLD, COPD, cirrhosis, diabetes, DLD, epilepsy, ESRD, thrombophilia, HTN, MI, migraine, mood disorder, cancer, PVD, PFO, rheumatological disease, syncope, thrombectomy, HD, stroke	Age Hb BMI Thrombectomy PLT LDL Creatinine CHF
Abujaber et al. [14]	19	NIHSS score, age, pneumonia, pre-stroke mRS, LOS, UTI, stroke-type, sex, ethnicity, BMI, diabetes, HTN, DLD, stroke, AF, CAD, CHF, smoking, admission location	Pneumonia LOS Stroke type NIHSS score
Bernardini et al. [15]	68	Sex, age, paroxysmal AF, HTN, diabetes, CHF, CAD, PVD, COPD, cancer, dementia, anemia, falls, previous bleeds, stroke, eGFR, antiplatelet drugs, CHAD2DS2VASc score, HAS-BLED score, DOACs, VKAs, creatinine, RBC	Hb (−0.8–1.4) Creatinine (−0.8–0.5) RBC (−0.5–0.5) eGFR (−0.6–0.6) BMI (−0.5–0.6) PLT (−1.2–1) Age (−0.5–0.5) Previous bleeds (0–1.9)
Bisson et al. [33]	16	Age, sex, HTN, diabetes, CHF, PVD, CHAD2DS2VASc score, SE, CAD, obesity, CKD, CLD, anemia, COPD, cancer, inflammatory disease, alcohol, thyroid disease, DLD, valvular disease, smoking	Age HTN CHF CKD Anemia COPD Thyroid disease DLD
Chen et al. [20]	12	CKD, age, HTN, AF treatment, elevated AST, diuretic drugs, sex, diabetes, prior stroke/TIA/SE, enlarged LA, AF, mitral valve	CKD (0.049) Age (0.046) HTN (0.045) AF treatment (0.04) Elevated AST (0.035) Diuretic drugs (0.032)
Colangelo et al. [21]	13	Previous stroke, Barthel index, AF, DLD, age, diabetes, sex, glycemia, BMI, HTN, cholesterol, smoking, alcohol	Previous stroke Barthel index AF DLD Age Diabetes Sex
Gkantzios et al. [16]	32	Age, sex, stroke type, SBP, glucose, CRP, ESR, HTN, smoking, diabetes, DLD, AF, previous stroke, previous MI, CHF, heart valve history, alcohol, antiplatelet drugs, anticoagulant drugs, 72 h data (SBP, intubation, cholesterol, LDL, T3 thyroid hormone, respiratory infection), stroke localization, NIHSS score	Respiratory infection (−0.3–0.21) Intubation (−1.5–0.35) Smoking (−0.22–0.1) SBP (−0.15–0.05) NIHSS score (−0.28–0.18) Age (−0.15–0.08) Stroke localization (−0.28–0.9)
Goto et al. [17]	10	Sex, age, DMI, LVEF, AF type, CHF, CAD, ACS, CHAD2DS2VASc score, HAS-BLED score	N/A
Han et al. [22]	11	Age, sex, MI, HTN, CHF, diabetes, PVD, CAD, Charlson comorbidity index, Selim comorbidity score, CHAD2DS2VASc score	N/A
Handy et al. [23]	N/A	Age, age at AF diagnosis, follow-up time post AF diagnosis, sex, ethnicity	N/A
Jeon et al. [10]	43	Age, sex, BMI, initial clinical status (DBP, SBP, pulse rate, NIHSS score), pre-stroke mRS, CHAD2DS2VASc score, CHADS2 score, stroke onset time, stroke, AF, CHF, HTN, diabetes, CAD, PVD, imaging findings, laboratory findings (WBC, Hb, PLT, CRP, glucose, fasting glucose, HbA1c, cholesterol, TG, HDL, LDL, AST, ALT, ALP, bilirubin, uric acid, creatinine, CrCl, fibrinogen, homocysteine, CK-MB, FFA, thrombolytic treatment	NIHSS score (0.6) Imaging findings—DWI lesion pattern (0.51) Pre-stroke mRS (0.49) CRP (0.49) Age (0.42)
Jung et al. [18]	N/A	N/A	N/A
Jung et al. [24]	58	CHAD2DS2VASc score, age, sex, insurance type, income level, CHF, HTN, diabetes, PVD, surgical history, HLD, dorsopathies, vision loss, thyroid disease, arrhythmias, obesity, gout, prostatic hyperplasia CLD, COPD, gynecological problems, osteoporosis, CKD, PE, hearing loss, gallbladder/biliary tract/pancreatic disorders, hemorrhoids, diverticulitis, arthritis (OA, RA), heart valve disease, neuropathy, dizziness, incontinence, ureter stones, anemia, psoriasis, headache, Parkinson’s disease, cancer, allergy, chronic gastritis, sexual dysfunction, insomnia, hypotension, depression, somatoform disorder, dementia, anxiety, afloqualone drugs, smoking, exercise, alcohol	Age Sex Thyroid disease Arrhythmias Hemolytic anemia Cancer Hemorrhoids Diabetes HTN CKD CHF HLD PVD Gout PE Chronic gastritis
Choi et al. [35]	23	Fasting glucose, NIHSS score, mRS, initial glucose, lateralization of ischemic lesion, QRS axis, ALP, homocysteine, SVS, intracranial atherosclerosis, FDP = fibrin degradation product, LA diameter, DBP, D-dimer, hemorrhagic transformation, AST, hematocrit, uric acid, cholesterol, T-axis, bilirubin, LDL	Fasting glucose (0.14) NIHSS score (0.125) mRS (0.07) Initial glucose (0.06) Lateralization of ischemic lesion (0.045) QRS axis (0.045) ALP (0.044)
Li et al. [25]	167	N/A	N/A
Lip et al. [26]	19	Sex, age, CHF, HTN, diabetes, stroke, PVD, valvular disease, CAD, CKD, COPD, major bleeding, alcohol, alcohol disorders, inflammatory diseases, lipid disorders, CHADS2, CHAD2DS2VASc	N/A
Lu et al. [27]	43	Previous stroke/TIA/embolism, age, cardiology admission, year of cohort entry, Hb, length of hospital stay, rate control drugs, bleeding history, eGFR, anti-arrhythmic drugs, gastric protective agents, diabetes, PE, AF, antiplatelet therapy, HTN, heart failure, DOACs, depression, falls	Previous stroke/TIA/embolism (0.20) Age (0.135) Cardiology admission (0.11) Year of cohort entry (0.9) Hb (0.8) Length of hospital stay (0.06)
Ming et al. [5]	N/A	Peak mitral A-wave velocity, HDL, age, heart rate, LA volume, eGFR, SBP, mRS, cholesterol, sex, LDL, body surface area, triglycerides, BMI, DBP, height, NIHSS score	Peak mitral A-wave velocity (−0.5–1) HDL (−0.4–1.5) Age (−0.7–0.5) Heart rate (−0.65–0.55) LA volume (−0.5–0.8) eGFR (−0.4–0.5) SBP (−0.5–0.4)
Nishi et al. [28]	14	Age, height, weight, stroke, HTN, catheter ablation, antiplatelet drug, antiarrhythmic drug, creatinine, urea, mitral/aortic regurgitation, LV mass, relative wall thickness	N/A
Papadopoulou et al. [29]	99	Age, AF, height, weight, waist circumference, sex, no. of medications taken, albumin, aspirin, SBP, pulse rate, bilirubin, urate, seated height, FEV1, testosterone, cancer, hip circumference, triglycerides	Age (0.49) AF (0.23) Height (0.12) Weight (0.1) Waist circumference (0.08) Sex (0.07) No. of medications taken (0.06)
Rebollo et al. [30]	N/A	Previous stroke, age, antithrombotic drugs, HTN, BMI, lipid modifying agents, extrapyramidal disorders, gastritis/duodenitis, creatinine, corticosteroids, drugs for peptic ulcer and gastro-esophageal reflux disease, glucose	Previous stroke Age Antithrombotic drugs HTN BMI Lipid modifying agents Extrapyramidal disorders
Sung et al. [34]	20	Age, triglyceride, PLT, numbness, heart rate, pulse pressure, sex, creatinine, diabetes, urea, BMI	Age (0.5) Triglyceride (0.23) PLT (0.21) Numbness (0.2) Heart rate (0.18)
Truong et al. [31]	53	Age, sex, ethnicity, year, geographical region, household median income, percentage of household with education level below high school, HTN, CHF, diabetes, previous stroke, PVD, previous bleeding, CKD, CLD, alcohol, COPD, hematological disorders, dementia, depression, thrombocytopenia, peptic ulcer disease, cancer, AFib, cancer type, cancer stage, tumor grade, active cancer status, antiplatelet drugs, anti-inflammatory drugs, ACE inhibitors, ARBs, calcium channel blockers, beta blockers, antiarrhythmic medications, diuretics, statins, pump proton inhibitors, serotonin reuptake inhibitors	No high school education Household medium income Time from cancer diagnosis Previous stroke ARBs Anti-inflammatory drugs Hematological disorder
Zhang et al. [32]	N/A	N/A	N/A

Abbreviations: BMI: body mass index; BP: blood pressure; DBP: diastolic blood pressure; SBP: systolic blood pressure; Hb: hemoglobin; LDL: low-density lipoprotein; HDL: high-density lipoprotein; PLT: platelet count; tPA: alteplase; CHF: congestive heart failure; CKD: chronic kidney disease; CLD: chronic liver disease; COPD: chronic obstructive pulmonary disease; DLD: dyslipidemia; ESRD: end stage renal disease; HTN: hypertension; MI: myocardial Infarction; PVD: peripheral vascular disease; PFO: patent foramen ovale; HD: heart disease; mRS: modified Rankin Scale; LOS: length of stay; UTI: urinary tract infection; CAD: coronary artery disease; eGFR: estimated glomerular filtration rate; CHAD2DS2VASc: congestive heart failure, hypertension, age ≥75 (doubled), diabetes, stroke (doubled), vascular disease, age 65 to 74, and sex category (female); HAS-BLED: hypertension, abnormal renal/liver function, stroke, bleeding history or predisposition, labile INR, elderly, drugs/alcohol concomitantly; FDP: fibrin degradation product.

## Data Availability

The original contributions presented in the study are included in the article and online supplemental information, and further inquiries can be directed to the corresponding author.

## Supplementary Materials

The following supporting information can be downloaded at: https://www.mdpi.com/article/10.3390/diagnostics14212391/s1, Search Strategy; Table S1: Preferred Reporting Items for Systematic Reviews and Meta-Analyses (PRISMA) 2020 checklist. Table S2: Meta-analysis of Observational Studies in Epidemiology (MOOSE) checklist. Table S3: STARD-2015 Checklist for Diagnostic Accuracy Studies. Table S4: Methodological quality assessment of included studies using the modified Jadad scale and assessment of funding bias.
