# Supplementary material for: Evaluating Machine Learning Models for Stroke Prognosis and Prediction in Atrial Fibrillation Patients: A Comprehensive Meta-Analysis
Source: Diagnostics (Basel). 2024 Oct 26;14(21):2391. doi: 10.3390/diagnostics14212391 (PMC11545060; doi:10.3390/diagnostics14212391)
Supplement: Supplementary file 1 [file diagnostics-14-02391-s001.zip › diagnostics-3253018-supplementary.pdf]

Supplemental Information

Evaluating the Predictive Accuracy of Machine Learning in Acute Ischemic Stroke Management Among Atrial Fibrillation Patients: A Meta-Analysis

Table of Contents

	Page Number
**1. Search Strategy**	2
**2. Supplemental Tables**	3
2.1. Table S1: Preferred Reporting Items for Systematic Reviews and Meta-Analyses (PRISMA) 2020 checklist.	3
2.2. Table S2: Meta-analysis of Observational Studies in Epidemiology (MOOSE) checklist.	8
2.3. Table S3: STARD-2015 Checklist for Diagnostic Accuracy Studies.	10
2.4. Table S4: Methodological quality assessment of included studies using the modified Jadad scale and assessment of funding bias.	12

1. Search Strategy

Database/ sources	Search Strategy	Filters	Search Date	Hits
PubMed	("Atrial Fibrillation" [Mesh] OR "AF" [Mesh]) AND ("Machine Learning" [Mesh] OR "Artificial Intelligence" [Mesh] OR "Deep Learning" [Mesh]) AND ("Acute Ischemic Stroke" [Mesh] OR "Cerebrovascular Disorders" [Mesh]) AND ("Clinical Outcomes" [Mesh]) AND ("Prognosis" [Mesh]) AND ("Prediction" [Mesh])	**Article type:**<br>• Adaptive Clinical Trial<br>• Clinical Study (Clinical Trial, Clinical Trial Phase I, II, III, IV, clinical trial protocol)<br>• Multicenter study<br>• Observational study<br><br>**Publication date:**<br>• January 2019 - May 2024<br><br>**Species:**<br>• Human<br><br>**Article Language:**<br>• English<br><br>**Age:**<br>• Adult: 18+ years	26/05/2024	160
Embase	("Atrial Fibrillation" [Emtree] OR "AF" [Emtree]) AND ("Machine Learning" [Emtree] OR "Artificial Intelligence" [Emtree] OR "Deep Learning" [Emtree]) AND ("Acute Ischemic Stroke" [Emtree] OR "Cerebrovascular Disorders" [Emtree]) AND ("Clinical Outcomes" [Emtree]) AND ("Prognosis" [Emtree]) AND ("Prediction" [Emtree])	**Limits:**<br>• Human<br><br>**Publication year:**<br>• 2019 – Current<br><br>**Languages:**<br>• English<br><br>**Human Age Group:**<br>• Adult: 18 to 64<br>• Adult: 65+ years	28/05/2024	100
Cochrane	(Atrial Fibrillation AND Machine Learning) AND (Ischemic Stroke AND Clinical Outcomes) AND (Prediction AND Prognosis)	**Date Published:**<br>• January 2019 - May 2024	01/06/2024	50
Google Scholar	Machine learning, Acute Ischemic Stroke, Atrial Fibrillation, Prognosis, Prediction	**Date Published:**<br>• January 2019 - May 2024	02/06/2024	55

2. Supplementary Tables

Table S1. Preferred Reporting Items for Systematic Reviews and Meta-Analyses (PRISMA) 2020 checklist.

Section and Topic	Item #	Checklist item	Location where item is reported (page)
**TITLE**			
Title	1	Identify the report as a systematic review.	1
**ABSTRACT**			
Abstract	2	See the PRISMA 2020 for Abstracts checklist.	2
**INTRODUCTION**			
Rationale	3	Describe the rationale for the review in the context of existing knowledge.	3
Objectives	4	Provide an explicit statement of the objective(s) or question(s) the review addresses.	3
**METHODS**			
Eligibility criteria	5	Specify the inclusion and exclusion criteria for the review and how studies were grouped for the syntheses.	4
Information sources	6	Specify all databases, registers, websites, organisations, reference lists and other sources searched or consulted to identify studies. Specify the date when each source was last searched or consulted.	4
Search strategy	7	Present the full search strategies for all databases, registers, and websites, including any filters and limits used.	4, Figure 1
Selection process	8	Specify the methods used to decide whether a study met the inclusion criteria of the review, including how many reviewers screened each record and each report retrieved, whether they worked independently, and if applicable, details of automation tools used in the process.	4
Data collection process	9	Specify the methods used to collect data from reports, including how many reviewers collected data from each report, whether they worked independently, any	4-5

Section and Topic	Item #	Checklist item	Location where item is reported (page)
		processes for obtaining or confirming data from study investigators, and if applicable, details of automation tools used in the process.	
Data items	10a	List and define all outcomes for which data were sought. Specify whether all results that were compatible with each outcome domain in each study were sought (e.g., for all measures, time points, analyses), and if not, the methods used to decide which results to collect.	4
	10b	List and define all other variables for which data were sought (e.g., participant and intervention characteristics, funding sources). Describe any assumptions made about any missing or unclear information.	4
Study risk of bias assessment	11	Specify the methods used to assess risk of bias in the included studies, including details of the tool(s) used, how many reviewers assessed each study and whether they worked independently, and if applicable, details of automation tools used in the process.	4,5
Effect measures	12	Specify for each outcome the effect measure(s) (e.g. risk ratio, mean difference) used in the synthesis or presentation of results.	5
Synthesis methods	13a	Describe the processes used to decide which studies were eligible for each synthesis (e.g., tabulating the study intervention characteristics and comparing against the planned groups for each synthesis (item #5)).	4
	13b	Describe any methods required to prepare the data for presentation or synthesis, such as handling of missing summary statistics, or data conversions.	5
	13c	Describe any methods used to tabulate or visually display results of individual studies and syntheses.	N/A
	13d	Describe any methods used to synthesize results and provide a rationale for the choice(s). If meta-analysis was performed, describe the model(s), method(s) to identify	4,5

Section and Topic	Item #	Checklist item	Location where item is reported (page)
		the presence and extent of statistical heterogeneity, and software package(s) used.	
	13e	Describe any methods used to explore possible causes of heterogeneity among study results (e.g., subgroup analysis, meta-regression).	6
	13f	Describe any sensitivity analyses conducted to assess robustness of the synthesized results.	5
Reporting bias assessment	14	Describe any methods used to assess risk of bias due to missing results in a synthesis (arising from reporting biases).	5
Certainty assessment	15	Describe any methods used to assess certainty (or confidence) in the body of evidence for an outcome.	5,6
**RESULTS**			
Study selection	16a	Describe the results of the search and selection process, from the number of records identified in the search to the number of studies included in the review, ideally using a flow diagram.	4, Figure 1
	16b	Cite studies that might appear to meet the inclusion criteria, but which were excluded, and explain why they were excluded.	N/A
Study characteristics	17	Cite each included study and present its characteristics.	Table 1-7, mentioned in pages 6-9
Risk of bias in studies	18	Present assessments of risk of bias for each included study.	N/A
Results of individual studies	19	For all outcomes, present, for each study: (a) summary statistics for each group (where appropriate) and (b) an effect estimate and its precision (e.g. confidence/credible interval), ideally using structured tables or plots.	6-9
Results of syntheses	20a	For each synthesis, briefly summarise the characteristics and risk of bias among contributing studies.	6-9
	20b	Present results of all statistical syntheses conducted. If	6-9

Section and Topic	Item #	Checklist item	Location where item is reported (page)
		meta-analysis was done, present for each the summary estimate and its precision (e.g., confidence/credible interval) and measures of statistical heterogeneity. If comparing groups, describe the direction of the effect.	
	20c	Present results of all investigations of possible causes of heterogeneity among study results.	6-9
	20d	Present results of all sensitivity analyses conducted to assess the robustness of the synthesized results.	6-9
Reporting biases	21	Present assessments of risk of bias due to missing results (arising from reporting biases) for each synthesis assessed.	N/A
Certainty of evidence	22	Present assessments of certainty (or confidence) in the body of evidence for each outcome assessed.	6-9
**DISCUSSION**			
Discussion	23a	Provide a general interpretation of the results in the context of other evidence.	10-11
	23b	Discuss any limitations of the evidence included in the review.	11-12
	23c	Discuss any limitations of the review processes used.	12
	23d	Discuss implications of the results for practice, policy, and future research.	12
**OTHER INFORMATION**			
Registration and protocol	24a	Provide registration information for the review, including register name and registration number, or state that the review was not registered.	4
	24b	Indicate where the review protocol can be accessed, or state that a protocol was not prepared.	N/A
	24c	Describe and explain any amendments to information provided at registration or in the protocol.	N/A
Support	25	Describe sources of financial or non-financial support for the review, and the role of the funders or sponsors in the	13-14

Section and Topic	Item #	Checklist item	Location where item is reported (page)
		review.	
Competing interests	26	Declare any competing interests of review authors.	14
Availability of data, code, and other materials	27	Report which of the following are publicly available and where they can be found: template data collection forms; data extracted from included studies; data used for all analyses; analytic code; any other materials used in the review.	N/A

Table S2. Meta-analysis of Observational Studies in Epidemiology (MOOSE) checklist.

Item Number	Recommendation	Reported on Page Number
Reporting of background should include		
1	Problem definition	3
2	Hypothesis statement	N/A
3	Description of study outcome(s)	6-9
4	Type of exposure or intervention used	4-5
5	Type of study designs used	4-5
6	Study population	6-9
Reporting of search strategy should include		
7	Qualifications of searchers (e.g., librarians and investigators)	N/A
8	Search strategy, including time period included in the synthesis and key words	3, Figure 1
9	Effort to include all available studies, including contact with authors	N/A
10	Databases and registries searched	4
11	Search software used, name and version, including special features used (e.g., explosion)	5
12	Use of hand searching (e.g., reference lists of obtained articles)	38-40, References
13	List of citations located and those excluded, including justification	N/A
14	Method of addressing articles published in languages other than English	N/A
15	Method of handling abstracts and unpublished studies	5
16	Description of any contact with authors	N/A
Reporting of methods should include		
17	Description of relevance or appropriateness of studies assembled for assessing the hypothesis to be tested	N/A
18	Rationale for the selection and coding of data (e.g., sound clinical principles or convenience)	5-6
19	Documentation of how data were classified and coded (e.g., multiple raters, blinding and interrater reliability)	N/A

20	Assessment of confounding (e.g., comparability of cases and controls in studies where appropriate)	N/A
21	Assessment of study quality, including blinding of quality assessors, stratification, or regression on possible predictors of study results	Supplementary information
22	Assessment of heterogeneity	8-9
23	Description of statistical methods (e.g., complete description of fixed or random effects models, justification of whether the chosen models account for predictors of study results, dose-response models, or cumulative meta-analysis) in sufficient detail to be replicated	N/A
24	Provision of appropriate tables and graphics	4 (Figure 1), 6-9 (Tables 1-7)
Reporting of results should include		
25	Graphic summarizing individual study estimates and overall estimates.	6-9 (Tables 1-7)
26	Table giving descriptive information for each study included	6-9 (Tables 1-7)
27	Results of sensitivity testing (e.g., subgroup analysis)	6-9 (Tables 1-7)
Reporting of discussion should include		
29	Quantitative assessment of bias (e.g., publication bias)	12
30	Justification for exclusion (e.g., exclusion of non-English language citations)	4
31	Assessment of quality of included studies	Supplementary information
Reporting of conclusions should include		
32	Consideration of alternative explanations for observed results	10-11
33	Generalization of the conclusions (i.e., appropriate for the data presented and within the domain of the literature review)	13
34	Guidelines for future research	12-13
35	Disclosure of funding source	13

Table S3: STARD-2015 Checklist for Diagnostic Accuracy Studies.

Section & Topic	No	Item	Reported on page
**TITLE OR ABSTRACT**			
	**1**	Identification as a study of diagnostic accuracy using at least one measure of accuracy.<br>(such as sensitivity, specificity, predictive values, or AUC)	7,8
**ABSTRACT**			
	**2**	Structured summary of study design, methods, results, and conclusions<br>(for specific guidance, see STARD for Abstracts)	2
**INTRODUCTION**			
	**3**	Scientific and clinical background, including the intended use and clinical role of the index test	3
	**4**	Study objectives and hypotheses	3
**METHODS**			
Study design	**5**	Whether data collection was planned before the index test and reference standard<br>were performed (prospective study) or after (retrospective study)	4-5
Participants	**6**	Eligibility criteria	4
	**7**	On what basis potentially eligible participants were identified<br>(such as symptoms, results from previous tests, inclusion in registry)	4
	**8**	Where and when potentially eligible participants were identified<br>(setting, location, and dates)	N/A
	**9**	Whether participants formed a consecutive, random or convenience series	N/A
Test methods	**10a**	Index test, in sufficient detail to allow replication	N/A
	**10b**	Reference standard, in sufficient detail to allow replication	N/A
	**11**	Rationale for choosing the reference standard (if alternatives exist)	N/A
	**12a**	Definition of and rationale for test positivity cut-offs or result categories of the index test, distinguishing pre-specified from exploratory	N/A
	**12b**	Definition of and rationale for test positivity cut-offs or result categories of the reference standard, distinguishing pre-specified from exploratory	N/A
	**13a**	Whether clinical information and reference standard results were available<br>to the performers/readers of the index test	N/A
	**13b**	Whether clinical information and index test results were available<br>to the assessors of the reference standard	N/A
Analysis	**14**	Methods for estimating or comparing measures of diagnostic accuracy	5
	**15**	How indeterminate index test or reference standard results were handled	N/A
	**16**	How the missing data on the index test and reference standard were handled	N/A
	**17**	Any analyses of variability in diagnostic accuracy, distinguishing pre-specified from exploratory	N/A
	**18**	Intended sample size and how it was determined	N/A
**RESULTS**			
Participants	**19**	Flow of participants, using a diagram	N/A
	**20**	Baseline demographic and clinical characteristics of participants	6-9
	**21a**	Distribution of severity of disease in those with the target condition	N/A
	**21b**	Distribution of alternative diagnoses in those without the target condition	N/A
	**22**	Time interval and any clinical interventions between index test and reference standard	N/A
Test results	**23**	Cross tabulation of the index test results (or their distribution)<br>by the results of the reference standard	6-9

	**24**	Estimates of diagnostic accuracy and their precision (such as 95% confidence intervals)	N/A
	**25**	Any adverse events from performing the index test or the reference standard	N/A
**DISCUSSION**			
	**26**	Study limitations, including sources of potential bias, statistical uncertainty, and generalisability	11-12
	**27**	Implications for practice, including the intended use and clinical role of the index test	12-13
**OTHER INFORMATION**			
	**28**	Registration number and name of registry	4
	**29**	Where the full study protocol can be accessed	N/A
	**30**	Sources of funding and other support; role of funders	13

Table S4. Methodological quality assessment of included studies using the modified Jadad scale and assessment of funding bias.

Abbreviations: MJA = Modified Jadad Analysis

Note: For all criteria no = 0, yes = 1.

[a]: Criteria 1: Was the study randomized?

[b]: Criteria 2: Was the method of randomization appropriate?

[c]: Criteria 3: Was the study described as being blinded?

[d]: Criteria 4: Was the method of blinding appropriate? (Single or partially blinded = 0.5)

[e]: Criteria 5: Was there a description of withdrawals and dropouts?

[f]: Criteria 6: Was there a clear description of the inclusion/exclusion criteria?

[g]: Criteria 7: Was the method used to assess adverse events described?

[h]: Criteria 8: Was the method of statistical analysis described?

[i]: Total score = sum of scores across criteria 1-8

[j]: Funding bias: 0 = low potential for bias, 1-2 = moderate potential for bias (conflicts of interest and/or study received funding from corporations in the industry), 3 = high potential for bias (conflicts of interest and industry funding that had a high likelihood of interfering with the study)

Author	Criteria 1[a]	Criteria 2[b]	Criteria 3[c]	Criteria 4[d]	Criteria 5[e]	Criteria 6[f]	Criteria 7[g]	Criteria 8[h]	Total MJA Score[i]	Funding Bias[j]
Abedi et al. [1]	1	1	0	0	0	1	0	1	**4**	0
Abujaber et al. [2]	0	0	0	0	1	1	0	1	**3**	0
Bernardini et al. [3]	-	-	-	-	-	-	-	-	**-**	-
Bisson et al. [4]	1	1	0	0	1	0	0	1	**4**	0
Chen et al. [5]	1	1	0	0	1	1	0	1	**5**	0
Colangelo et al. [6]	0	0	0	0	1	1	0	1	**3**	0
Gkantzios et al. [7]	0	0	0	0	0	0	0	1	**1**	0
Goto et al. [8]	1	1	0	0	0	1	1	1	**5**	0
Han et al. [9]	1	1	0	0	1	1	0	1	**5**	0
Handy et al. [10]	1	1	0	0	0	1	0	1	**4**	0
Jeon et al. [11]	1	1	0	0	1	1	0	1	**5**	0
Jung et al. [12]	-	-	-	-	-	-	-	-	**-**	-
Jung et al. [13]	1	1	0	0	0	0	0	1	**3**	0
Choi et al. [14]	1	1	0	0	1	1	0	1	**5**	0
Li et al. [15]	1	1	0	0	0	0	0	1	**3**	0
Lip et al. [16]	1	1	0	0	0	0	0	1	**3**	0
Lu et al. [17]	1	1	0	0	1	1	0	1	**5**	0
Ming et al. [18]	1	1	0	0	0	0	0	1	**3**	0

Nishi et al. [19]	1	1	0	0	1	1	0	1	**5**	0
Papadopoulou et al. [20]	1	1	0	0	0	1	0	1	**4**	0
Rebollo et al. [21]	-	-	-	-	-	-	-	-	**-**	-
Sung et al. [22]	1	1	0	0	1	1	0	1	**5**	0
Truong et al. [23]	1	1	0	0	0	1	1	1	**5**	0
Zhang et al. [24]	1	1	0	0	0	1	0	1	**4**	0

References

1. Abedi, V.; Avula, V.; Razavi, S.M.; Bavishi, S.; Chaudhary, D.; Shahjouei, S.; Wang, M.; Griessenauer, C.J.; Li, J.; Zand, R. Predicting short and long-term mortality after acute ischemic stroke using EHR. *Journal of the Neurological Sciences* **2021**, *427(no pagination)*, doi:https://dx.doi.org/10.1016/j.jns.2021.117560.
2. Abujaber, A.A.; Alkhawaldeh, I.M.; Imam, Y.; Nashwan, A.J.; Akhtar, N.; Own, A.; Tarawneh, A.S.; Hassanat, A.B. Predicting 90-day prognosis for patients with stroke: a machine learning approach. *Frontiers in Neurology* **2023**, *14(no pagination)*, doi:https://dx.doi.org/10.3389/fneur.2023.1270767.
3. Bernardini, A.; Bindini, L.; Antonucci, E.; Berteotti, M.; Giusti, B.; Testa, S.; Palareti, G.; Poli, D.; Frasconi, P.; Marcucci, R. Machine learning approach for prediction of outcomes in anticoagulated patients with atrial fibrillation. *International Journal of Cardiology* **2024**, *407(no pagination)*, doi:https://dx.doi.org/10.1016/j.ijcard.2024.132088.
4. Bisson, A.; Lemrini, Y.; El-Bouri, W.; Bodin, A.; Angoulvant, D.; Lip, G.Y.H.; Fauchier, L. Prediction of incident atrial fibrillation in post-stroke patients using machine learning: a French nationwide study. *Clinical Research in Cardiology* **2023**, *112(6)*, 815-823, doi:https://dx.doi.org/10.1007/s00392-022-02140-w.
5. Chen, Y.; Gue, Y.; Calvert, P.; Gupta, D.; McDowell, G.; Azariah, J.L.; Namboodiri, N.; Bucci, T.; Jabir, A.; Tse, H.F.; et al. Predicting stroke in Asian patients with atrial fibrillation using machine learning: A report from the KERALA-AF registry, with external validation in the APHRS-AF registry. *Current Problems in Cardiology* **2024**, *49(4) (no pagination)*, doi:https://dx.doi.org/10.1016/j.cpcardiol.2024.102456.
6. Colangelo, G.; Ribo, M.; Montiel, E.; Dominguez, D.; Olive-Gadea, M.; Muchada, M.; Garcia-Tornel, A.; Requena, M.; Pagola, J.; Juega, J.; et al. PRERISK: A Personalized, Artificial Intelligence-Based and Statistically-Based Stroke Recurrence Predictor for Recurrent Stroke. *Stroke* **2024**, *55(5)*, 1200-1209, doi:https://dx.doi.org/10.1161/STROKEAHA.123.043691.
7. Gkantzios, A.; Kokkotis, C.; Tsiptsios, D.; Moustakidis, S.; Gkartzonika, E.; Avramidis, T.; Tripsianis, G.; Iliopoulos, I.; Aggelousis, N.; Vadikolias, K. From Admission to Discharge: Predicting National Institutes of Health Stroke Scale Progression in Stroke Patients Using Biomarkers and Explainable Machine Learning. *Journal of Personalized Medicine* **2023**, *13(9) (no pagination)*, doi:https://dx.doi.org/10.3390/jpm13091375.
8. Goto, S.; Goto, S.; Pieper, K.S.; Bassand, J.P.; Camm, A.J.; Fitzmaurice, D.A.; Goldhaber, S.Z.; Haas, S.; Parkhomenko, A.; Oto, A.; et al. New artificial intelligence prediction model using serial prothrombin time international normalized ratio measurements in atrial fibrillation patients on vitamin K antagonists: GARFIELD-AF. *Eur Heart J Cardiovasc Pharmacother* **2020**, *6*, 301-309, doi:10.1093/ehjcvp/pvz076.
9. Han, L.; Askari, M.; Altman, R.B.; Schmitt, S.K.; Fan, J.; Bentley, J.P.; Narayan, S.M.; Turakhia, M.P. Atrial fibrillation burden signature and near-term prediction of stroke: A machine learning analysis. *Circulation: Cardiovascular Quality and Outcomes* **2019**, *12(10) (no pagination)*, doi:https://dx.doi.org/10.1161/CIRCOUTCOMES.118.005595.
10. Handy, A.; Wood, A.; Sudlow, C.; Tomlinson, C.; Kee, F.; Thygesen, J.H.; Mohammad, M.; Sofat, R.; Dobson, R.; Ip, S.; et al. A nationwide deep learning pipeline to predict stroke and COVID-19 death in atrial fibrillation. *medRxiv.* **2021**, *21*, doi:https://dx.doi.org/10.1101/2021.12.20.21268113.
11. Jeon, E.T.; Jung, S.J.; Yeo, T.Y.; Seo, W.K.; Jung, J.M. Predicting short-term outcomes in atrial-fibrillation-related stroke using machine learning. *Frontiers in Neurology* **2023**, *14(no pagination)*, doi:https://dx.doi.org/10.3389/fneur.2023.1243700.
12. Jung, J.M.; Jeon, E.T. Outcome predictions using machine learning in atrial fibrillation-related stroke. *Circulation. Conference: American Hearts Association's* **2021**, *144*, doi:https://dx.doi.org/10.1161/circ.144.suppl-1.11932.
13. Jung, S.; Song, M.K.; Lee, E.; Bae, S.; Kim, Y.Y.; Lee, D.; Lee, M.J.; Yoo, S. Predicting Ischemic Stroke in Patients with Atrial Fibrillation Using Machine Learning.

Frontiers in bioscience (Landmark edition) **2022**, *27(3)*, 80, doi:https://dx.doi.org/10.31083/j.fbl2703080.
14. Choi, K.-H.; Kim, J.-H.; Kang, K.-W.; Kim, J.-T.; Choi, S.-M.; Lee, S.-H.; Park, M.-S.; Kim, B.-C.; Kim, M.-K.; Cho, K.-H. Impact of Microbleeds on Outcome Following Recanalization in Patients With Acute Ischemic Stroke. *Stroke* **2019**, *50*, 127-134, doi:doi:10.1161/STROKEAHA.118.023084.
15. Li, X.; Liu, H.; Du, X.; Zhang, P.; Hu, G.; Xie, G.; Guo, S.; Xu, M.; Xie, X. Integrated Machine Learning Approaches for Predicting Ischemic Stroke and Thromboembolism in Atrial Fibrillation. *AMIA Annu Symp Proc* **2016**, *2016*, 799-807.
16. Lip, G.Y.H.; Tran, G.; Genaidy, A.; Marroquin, P.; Estes, C.; Landsheft, J. Improving dynamic stroke risk prediction in non-Anticoagulated patients with and without atrial fibrillation: Comparing common clinical risk scores and machine learning algorithms. *European Heart Journal - Quality of Care and Clinical Outcomes* **2022**, *8(5)*, 548-556, doi:https://dx.doi.org/10.1093/ehjqcco/qcab037.
17. Lu, J.; Hutchens, R.; Hung, J.; Bennamoun, M.; McQuillan, B.; Briffa, T.; Sohel, F.; Murray, K.; Stewart, J.; Chow, B.; et al. Performance of multilabel machine learning models and risk stratification schemas for predicting stroke and bleeding risk in patients with non-valvular atrial fibrillation. *Computers in Biology and Medicine* **2022**, *150(no pagination)*, doi:https://dx.doi.org/10.1016/j.compbiomed.2022.106126.
18. Ming, C.; Lee, G.J.W.; Teo, Y.H.; Teo, Y.N.; Toh, E.M.S.; Li, T.Y.W.; Guo, C.Y.; Ding, J.; Zhou, X.; Teoh, H.L.; et al. Machine Learning Modeling to Predict Atrial Fibrillation Detection in Embolic Stroke of Undetermined Source Patients. *J Pers Med* **2024**, *14*, doi:10.3390/jpm14050534.
19. Nishi, H.; Oishi, N.; Ogawa, H.; Natsue, K.; Doi, K.; Kawakami, O.; Aoki, T.; Fukuda, S.; Akao, M.; Tsukahara, T. Predicting cerebral infarction in patients with atrial fibrillation using machine learning: The Fushimi AF registry. *Journal of Cerebral Blood Flow and Metabolism* **2022**, *42(5)*, 746-756, doi:https://dx.doi.org/10.1177/0271678X211063802.
20. Papadopoulou, A.; Harding, D.; Slabaugh, G.; Marouli, E.; Deloukas, P. Prediction of atrial fibrillation and stroke using machine learning models in UK Biobank. *medRxiv.* **2022**, *30*, doi:https://dx.doi.org/10.1101/2022.10.28.22281669.
21. Rebollo, P.; Wolk, A.; Luczko, M.; Tang, J.P. MSR124 Development of a Machine Learning Predictive Model for Stroke Among Patients With Non-Valvular Atrial Fibrillation Receiving Oral Anticoagulant Treatment. *Value in Health* **2022**, *25(12 Supplement)*, S374, doi:https://dx.doi.org/10.1016/j.jval.2022.09.1854.
22. Sung, S.F.; Sung, K.L.; Pan, R.C.; Lee, P.J.; Hu, Y.H. Automated risk assessment of newly detected atrial fibrillation poststroke from electronic health record data using machine learning and natural language processing. *Frontiers in Cardiovascular Medicine* **2022**, *9(no pagination)*, doi:https://dx.doi.org/10.3389/fcvm.2022.941237.
23. Truong, B.; Zheng, J.; Hornsby, L.; Fox, B.; Chou, C.; Qian, J. Development and Validation of Machine Learning Algorithms to Predict 1-Year Ischemic Stroke and Bleeding Events in Patients with Atrial Fibrillation and Cancer. *Cardiovascular Toxicology* **2024**, *24(4)*, 365-374, doi:https://dx.doi.org/10.1007/s12012-024-09843-8.
24. Zhang, F.; Zhang, Y.; Zhou, Q.; Shi, Y.; Gao, X.; Zhai, S.; Zhang, H. Using machine learning to identify proteomic and metabolomic signatures of stroke in atrial fibrillation. *Computers in Biology and Medicine* **2024**, *173(no pagination)*, doi:https://dx.doi.org/10.1016/j.compbiomed.2024.108375.
